# A Rare Presentation of Polygenic Inheritance Manifesting As Congenital Rubella Syndrome: A Case Report

**DOI:** 10.7759/cureus.32861

**Published:** 2022-12-23

**Authors:** Rishita Jain, Ashish Anjankar

**Affiliations:** 1 Medicine and Surgery, Jawaharlal Nehru Medical College, Datta Meghe Institute of Medical Sciences, Wardha, IND; 2 Biochemistry, Jawaharlal Nehru Medical College, Datta Meghe Institute of Medical Sciences, Wardha, IND

**Keywords:** chorioretinitis, pulmonary stenosis, autism, microcephaly, cyanosis, teratogen

## Abstract

Many traits and phenotypic characteristics present in the human body such as height, skin, pigmentation, hair, and eye color are inherited through many alleles present in different loci. This is known as polygenic inheritance. Congenital rubella syndrome (CRS) is a rare disease characterized by congenital deformities such as chorioretinitis and cataracts. This disease is endemic in India, and it is mainly caused by the rubella virus. We report a case of a 4.5-year-old female child who presented with breathlessness and radiolucent bone disease, for which she was already undergoing surgical interventions. The cell culture line was positive for rubella. The patient was treated with oxygen therapy via continuous positive airway pressure (CPAP) and had a moderate biventricular function.

## Introduction

First discovered by cell culture techniques in 1962, the rubella virus has a single, positive-sense RNA genome [1]. The rubella E1 protein binds to myelin oligodendrocyte that promotes in-vitro infection. This protein expresses itself ectopically on the sensory cells of the pregnant mother and gets transferred to the fetus through the placenta. The high similarity between myelin oligodendrocyte glycoprotein (MOG) and the rubella E2 protein and its capacity to demyelinate rat brain cells in response to rubella antibodies may explain the pathogenesis and vertical transmission of the virus from the mother to the fetus [2].

Infection with the rubella virus typically results in mild fever and rash in children and adults. Still, when it occurs during pregnancy, particularly in the first trimester, it can cause miscarriage, fetal death, stillbirth, or infants with congenital deformities, known as congenital rubella syndrome (CRS) [3]. Jaundice, hepatomegaly, splenomegaly, and rashes are the most prevalent systemic symptoms. All of the above-mentioned clinical manifestations associated with toxoplasmosis, rubella, cytomegalovirus, and human influenza virus (TORCH) group of infections are present in CRS; apart from these, chorioretinitis and cataracts are predominantly seen [4].

As many as 90% of the infected infants show signs of CRS when the disease strikes during the first 12 weeks of pregnancy. However, when infection occurs between 16 to 20 weeks, the likelihood of a symptomatic fetal infection drops to about 20%. After that, intrauterine transmission might happen, although the chance of CRS is minimal. Systemic and neurologic symptoms are the two main subtypes of the early clinical manifestations of congenital infections. In nations where women of childbearing age lack immunity to the illness, there is a higher risk of infection which can be prevented by vaccination or by immunoglobulin infusion. Four out of every 1000 live births were affected by CRS before the vaccine's release. The following signs and symptoms could manifest in all or some newborns with CRS: heart defects; issues with the eyes, such as glaucoma and cataracts; intellectual disabilities; microcephaly; sensorineural hearing loss; and growth restriction. Neuroimaging techniques such as CT, MRI, and ultrasound detect prenatal infections such as intracranial calcifications or white matter lesions [5]. Long-term sequelae include panencephalitis, insulin-dependent diabetes, thyroid disorders, and precocious puberty. Screening for rubella infection should be done during the first trimester of all pregnant females.

## Case presentation

A female child aged 4.5 years was brought by the mother with chief complaints of breathlessness and generalized swelling all over the body, especially over the face and trunk. As narrated by the mother, the child was apparently alright a few days before presentation when she started to notice swelling over the face which progressed toward the lower limb. This was treated by giving furosemide which is a loop diuretic (commonly sold under the brand name Lasix). The child also developed severe breathlessness for which she was given oxygen support. Further, the baby experienced a severe cold and cough that caused her respiratory distress eight months ago.

On physical examination, the child's birth history was an average of 2.5 kg, and no history of neonatal ICU stay. The child was afebrile with a pulse rate of 130 bpm and a respiratory rate of 28 to 30 breaths in a minute. Her oxygen saturation (sPO²) was 89%, and blood pressure was 92/58 mmHg. The anthropometry of the child is stated below in Table 1. 

**Table 1:** Anthropometric measurements of the patient

	Observed	Expected	Percentage
Height/length	90 cm	107 cm	84%
Weight	11 kg	17 kg	64%
Head circumference	47 cm	49.5 cm	95%

The child had achieved all the milestones as per age. She was immunized adequately as per the national immunization schedule. General appearance revealed deformity of the limbs and edema all over the body. The patient was afebrile on touch with no spikes, along with normal micturition and bowel movements. Systemic examination suggested bilateral pleural effusion in the lungs and respiratory distress, heart sounds were heard loudly, and ejection systolic murmur in the pulmonary area gradually led to pansystolic murmur. Abdominal examination suggested tenderness in the right hypochondrium, soft on palpation. The central nervous system (CNS) examination revealed that the child was conscious and active. Both kidneys were normal in size and shape, and echotexture with corticomedullary differentiation (CMD) was maintained. No focal lesion was noted, and no hydronephrosis or calculi were seen. The urinary bladder appeared normal with no ascites and no fluid seen in the peritoneal cavity.

**Figure 1:**
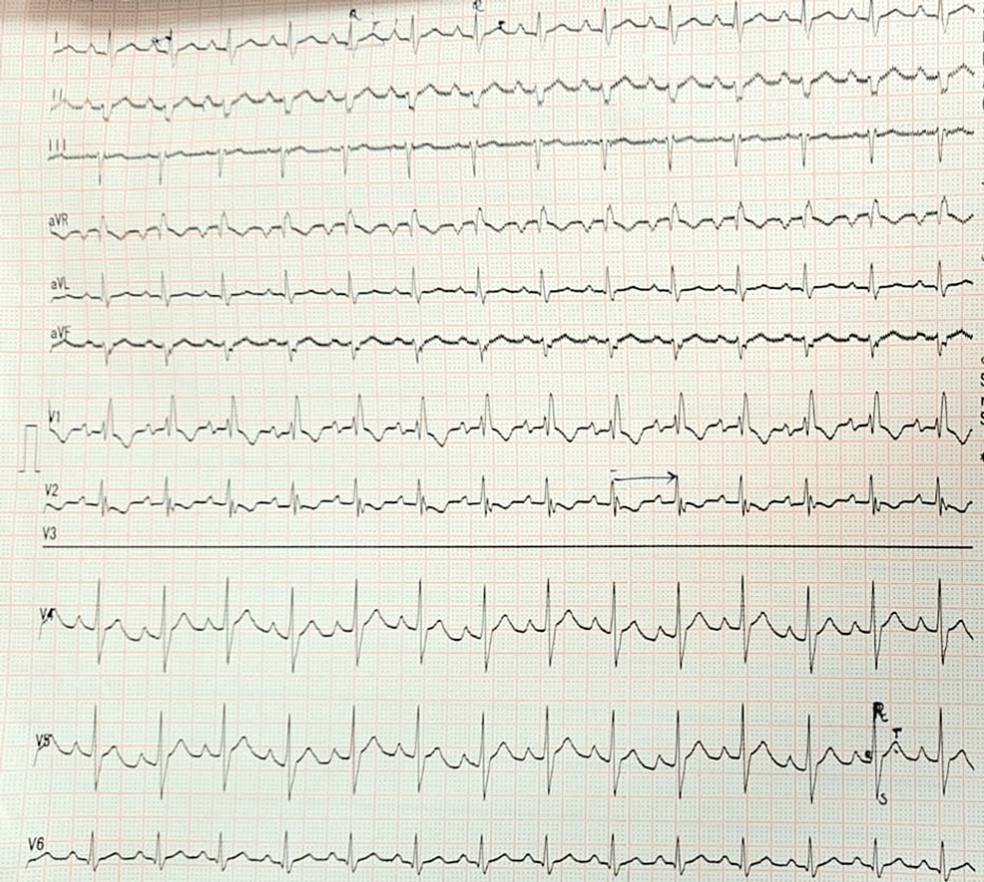
Electrocardiograph of the patient Severe left axis deviation with right atrium hypertrophy and sinus mode tachycardia

The electrocardiograph suggested levocardia, superior and inferior vena cava draining into the right side of the single atrium, and three pulmonary veins draining into the left side. Also indicated were dilated atrium with no interatrial septum, mild to moderate mitral regurgitation, mild tricuspid regurgitation, and normal size ventricles with normal biventricular function. There was a mild defect in the ventricular septum, standard semilunar valves, and pulmonary artery stenosis with patent ductus arteriosus. Pulmonary hypertension was also present. The patient also had radiolucent bone disease, for which she was already undergoing surgical interventions (Figure 1). 

On two-dimensional (2D) echocardiography, the patient was found to have an atrial septal defect, ventricular septal defect, patent ductus arteriosus, and pulmonary artery stenosis. The cell culture line was positive for the rubella virus. The patient had no diabetes, hypertension, bronchial asthma, or thyroid disorder history. Later follow-ups showed a precordial bulge with no dilated veins, scars, or sinuses. Palpatory examination revealed an apex beat at the fifth intercostal space at the anterior axillary line. Auscultatory analysis showed ejection systolic murmur (ESM) with loud P2 heard at the tricuspid area. Consistent saturation of oxygen was maintained by continuous positive airway pressure (CPAP) (~90%).

## Discussion

Humans are the only known reservoir of the rubella virus, with postnatal person-to-person transmission occurring via direct or airborne contact via respiratory secretions of infected persons. Although the early events surrounding infection are incompletely characterized, the virus almost certainly multiplies in the respiratory tract cells, which may also extend to the local lymph nodes and then undergoes viremic spread to target organs. Subsequent additional replication in selected target tissues, such as the spleen and lymph nodes, leads to a secondary viremia with a wide virus distribution. The virus can infect pregnant women who have not received the vaccination. However, it should be noted that not all pregnancies cause the vertical transfer of infection to the fetus.

Furthermore, not all rubella-infected fetuses develop congenital malformations. The respiratory exposure of a pregnant woman to the virus initiates the typical clinical course of CRS. The virus then spreads to the fetus through the fetal-placental barrier. The interruption of organogenesis during the embryonic period causes systemic inflammation in the fetus and several congenital abnormalities [6,7]. The periconceptional period and early pregnancy (eight to 10 weeks) are the most crucial and are the most significant risk of CRS, which is as high as 90%. Neonatal death may occur as a result of CRS. For infants with CRS hearing impairment, eye symptoms and developmental delay might not be detected in the early stages. Suppose maternal infection is diagnosed beyond 20 weeks of embryogenesis, in that case, the fetus might be infected. Still, it might not develop signs and symptoms of CRS. Infants with laboratory evidence of rubella and without any signs or symptoms of CRS are classified as having congenital rubella infection (CRI) only.

The clinical manifestations include congenital cataract, congenital sensorineural deafness, ventricular septal defect, tetralogy of Fallot, intellectual/learning difficulties, microcephaly, and intrauterine growth limitation. The serology markers are immunoglobulin (Ig)M and IgG levels [8]. The neurological manifestations include small head circumference, microcephaly, periventricular calcifications, white matter hypodensity, and ventriculomegaly. Progress toward the elimination of the virus in India has accelerated since 2012, and in 2020, rubella elimination was verified in approximately one-half of the countries in the world. The establishment of regional WHO rubella eradication targets to increase country commitment to elimination, and the availability of financial support from international partners to introduce rubella-containing vaccine (RCV) have contributed to the significant progress made toward eradication. Maternal rubella and CRS have no known treatments. Preventive measures become of utmost significance. All youngsters should receive vaccinations to stop epidemics. Children should receive a combination of vaccines against measles, mumps, and rubella at 15 months. Due to the possibility that vaccine-induced immunity would not last as long as naturally acquired protection, many authorities now advise that 10-year-olds receive a second dose of the rubella vaccination. Women of childbearing age who are susceptible to rubella and have antibodies against the disease in their blood should also receive the vaccination. The Centers for Disease Control and Prevention (CDC) advises women to wait three months after receiving a rubella immunization before planning their pregnancy [9].

## Conclusions

Our case describes an unusual manifestation of CRS. As the patient initially presented with breathlessness, immediate measures should be taken by providing oxygen therapy by CPAP. Regular tests such as complete blood count, C-reactive protein, kidney function test, liver function test, and electrocardiography are done to rule out differential diagnoses of other congenital diseases such as tetralogy of Fallot, Ebstein anomaly, and TORCH infections. As our patient had a ventricular septal defect, large atrial septal defect leading to common atria, regurgitation of both the AV valves, moderate pulmonary hypertension, and moderate biventricular function, surgical repair was performed after the patient was stabilized.

The medical management included antibiotics, ceftriaxone, pantop, Lasix, and Entomol. The basal metabolic index score of the patient was 2, with normal nutritional status and mild dementia. The pain score was 2 according to the pain scale assessment. After medication, the patient was temporarily stabilized with decreased respiratory distress, tachypnea, and tachycardia, due to which sucralfate syrup was given. There was no fever with spikes, and she had normal bowel and bladder motion. The nasopharyngeal tube maintained oxygen saturation. Further, on inspection, the precordial bulge was present but with no dilated veins or scars, or sinuses; on palpation, an apex beat was present at the fifth intercostal space in the anterior axillary line; on auscultation ESM with loud P2 was heard at the tricuspid area.
